# Supplementary figures and images for: Genome-wide identification and expression profiling of durian CYPome related to fruit ripening
Source: PLoS One. 2021 Nov 30;16(11):e0260665. doi: 10.1371/journal.pone.0260665 (PMC8631664; doi:10.1371/journal.pone.0260665)

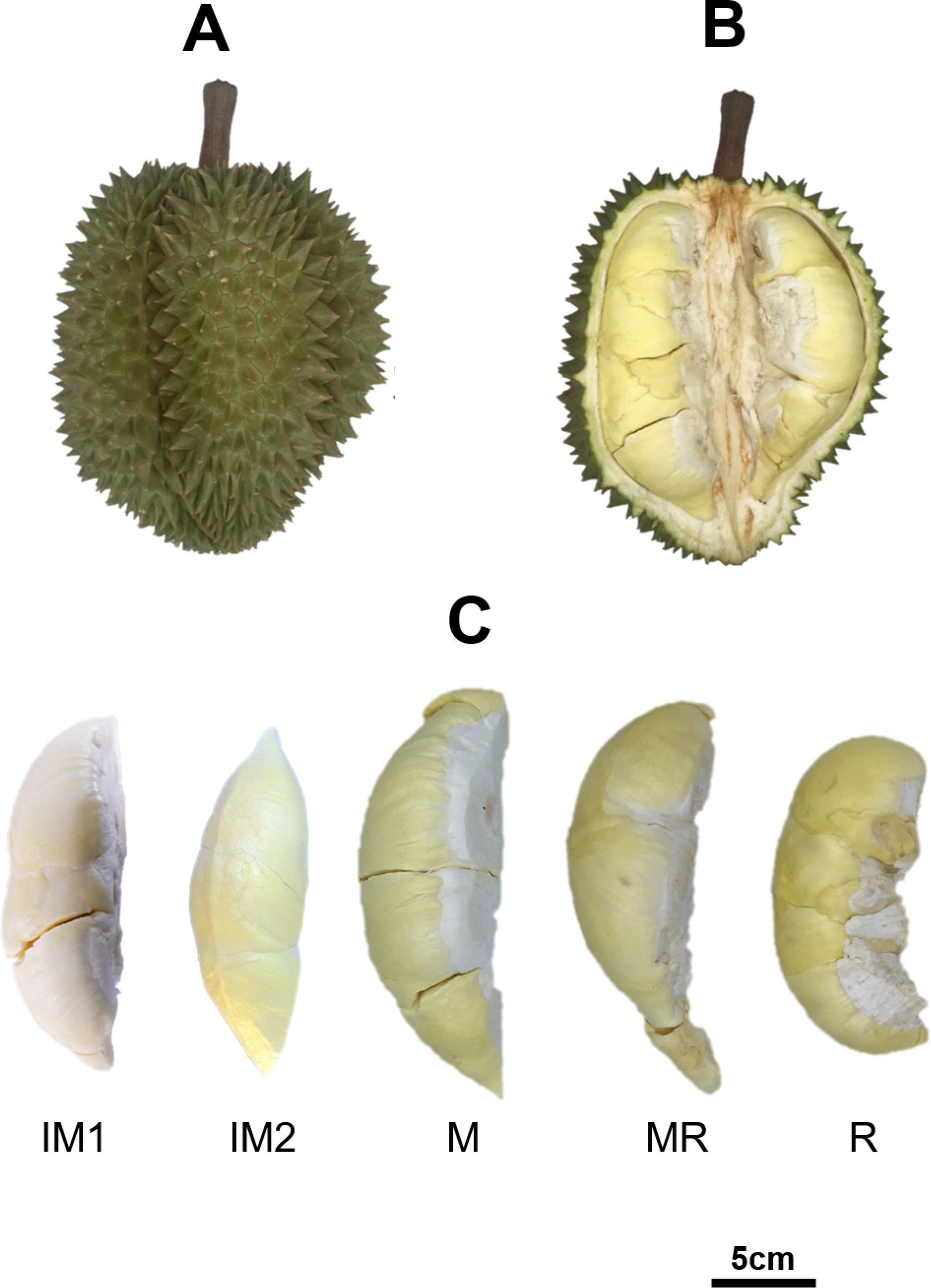

Supplement: S1 Fig — (A) whole fruit. (B) peeled fruit. (C) Arils across five developmental and ripening stages. Stage abbreviations: IM1, immature 1; IM2, immature 2; M, mature; MR, mid-ripe; R, ripe. (TIF) [file pone.0260665.s001.tif]

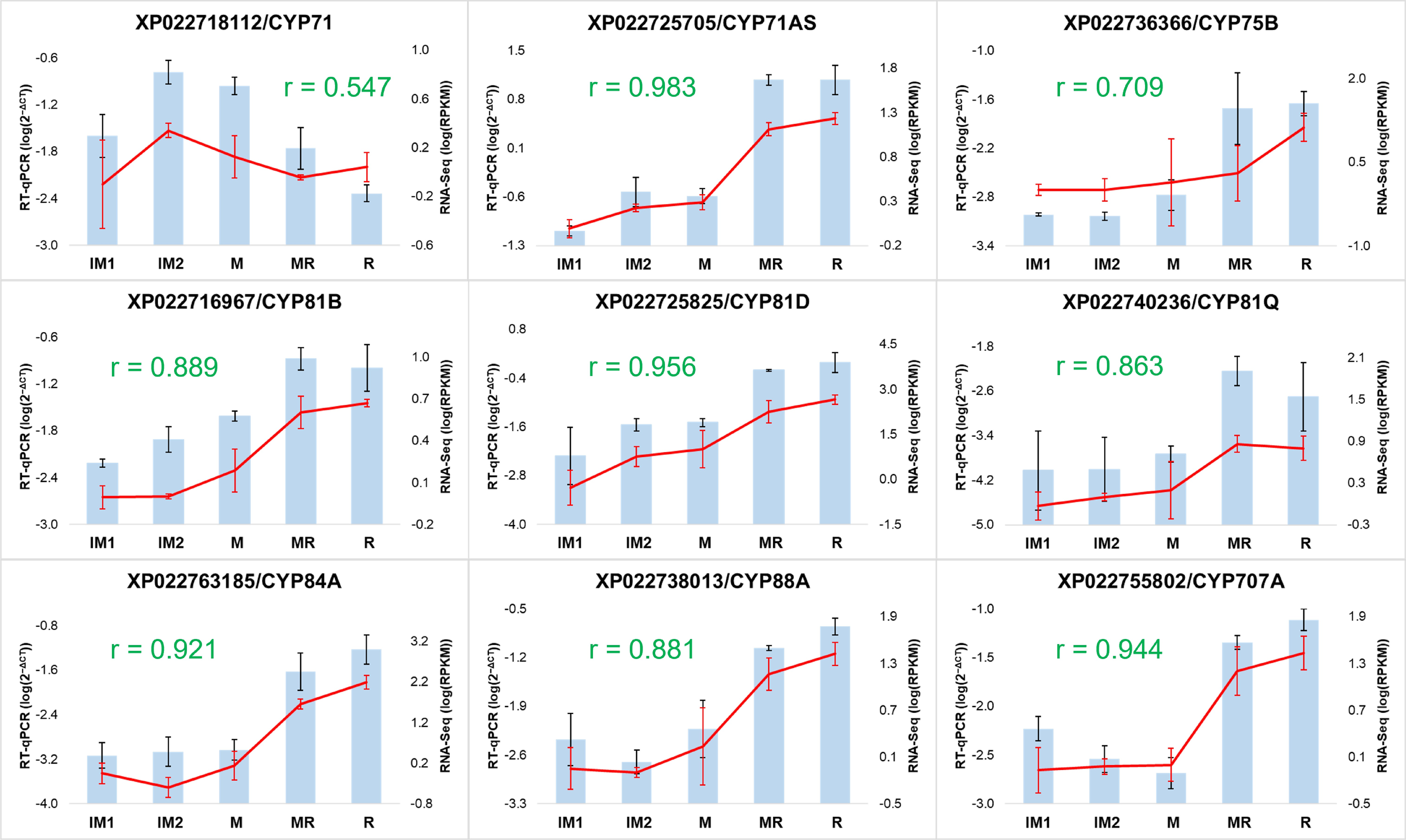

Supplement: S2 Fig — Protein ID followed by predicted P450 family are indicated above each chart. Blue bars and error bars represent the means and standard deviations of log(2−ΔCT) based on RT-qPCR. Red lines and error bars represent the means and standard deviations of log-RPKM values. Three biological replicates were used for each stage. Pearson correlation (r) values are shown in green letters (p = 0.05). Stage abbreviations: IM1, immature stage 1; IM2, immature stage 2; M, mature stage; MR, mid-ripe stage; R, ripe stage. (TIF) [file pone.0260665.s002.tif]

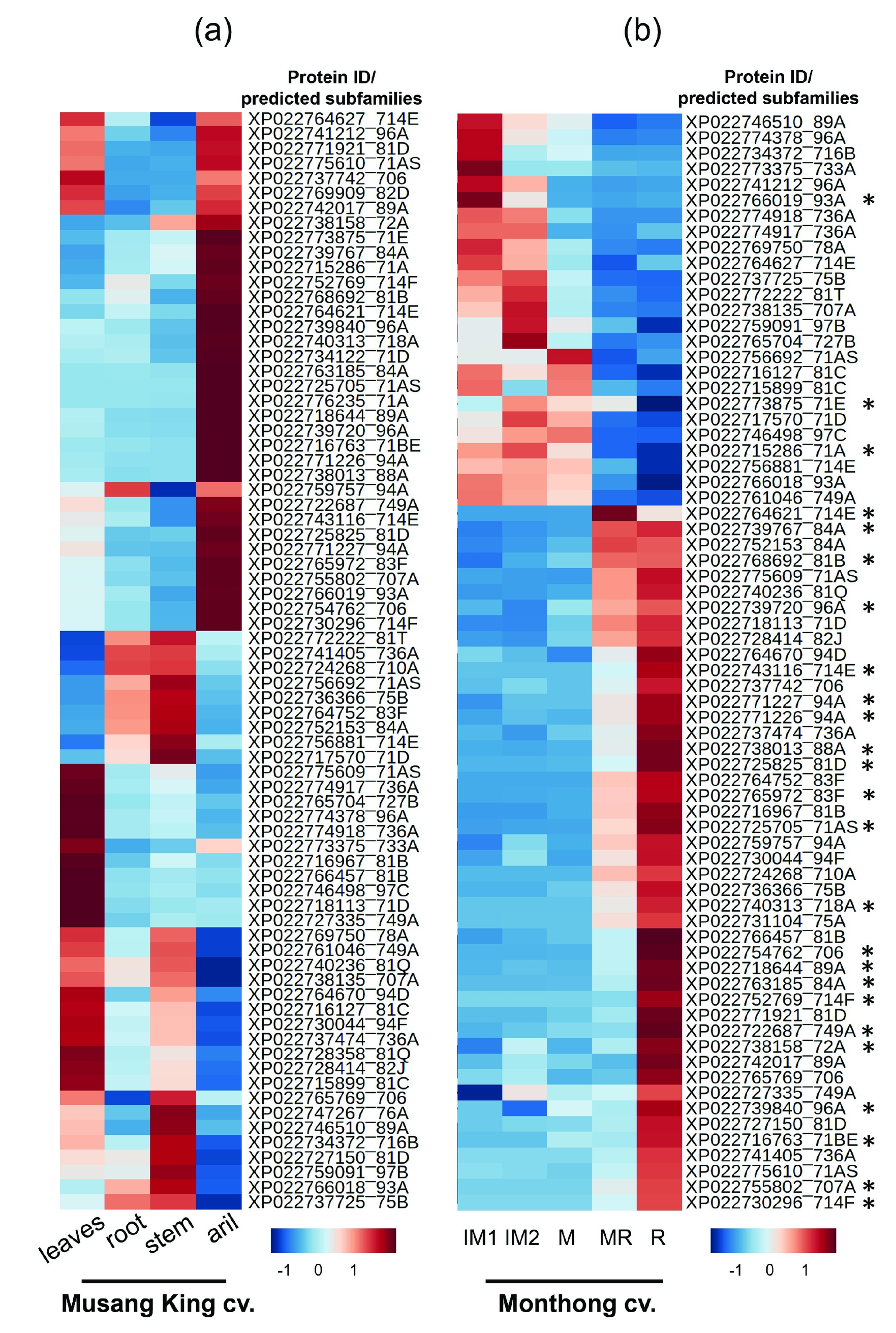

Supplement: S3 Fig — (A) Four tissues of Musangking cultivar. (B) Five ripening stages of Monthong cultivar. To simplify the heatmap, the top 70% ranked by partial least squares discriminant analysis (PLS-DA) and variable importance in projection (VIP) are shown and briefly categorized into two clusters, decreased and increased during the ripening stages. The heatmap was generated by MetaboAnalyst 5.0, an open-source R-based program. Data were sum normalized, log transformed, and auto scaled. Asterisks indicate fruit-specific P450s from the heatmap (a). The color key bars indicate the standard score (Z-score) of each gene expression level. Higher expression for each gene is presented in red; otherwise, blue was used. Stage abbreviations: IM1, immature1; IM2, immature2; M, mature; MR, mid-ripe; R, ripe. (TIF) [file pone.0260665.s003.tif]

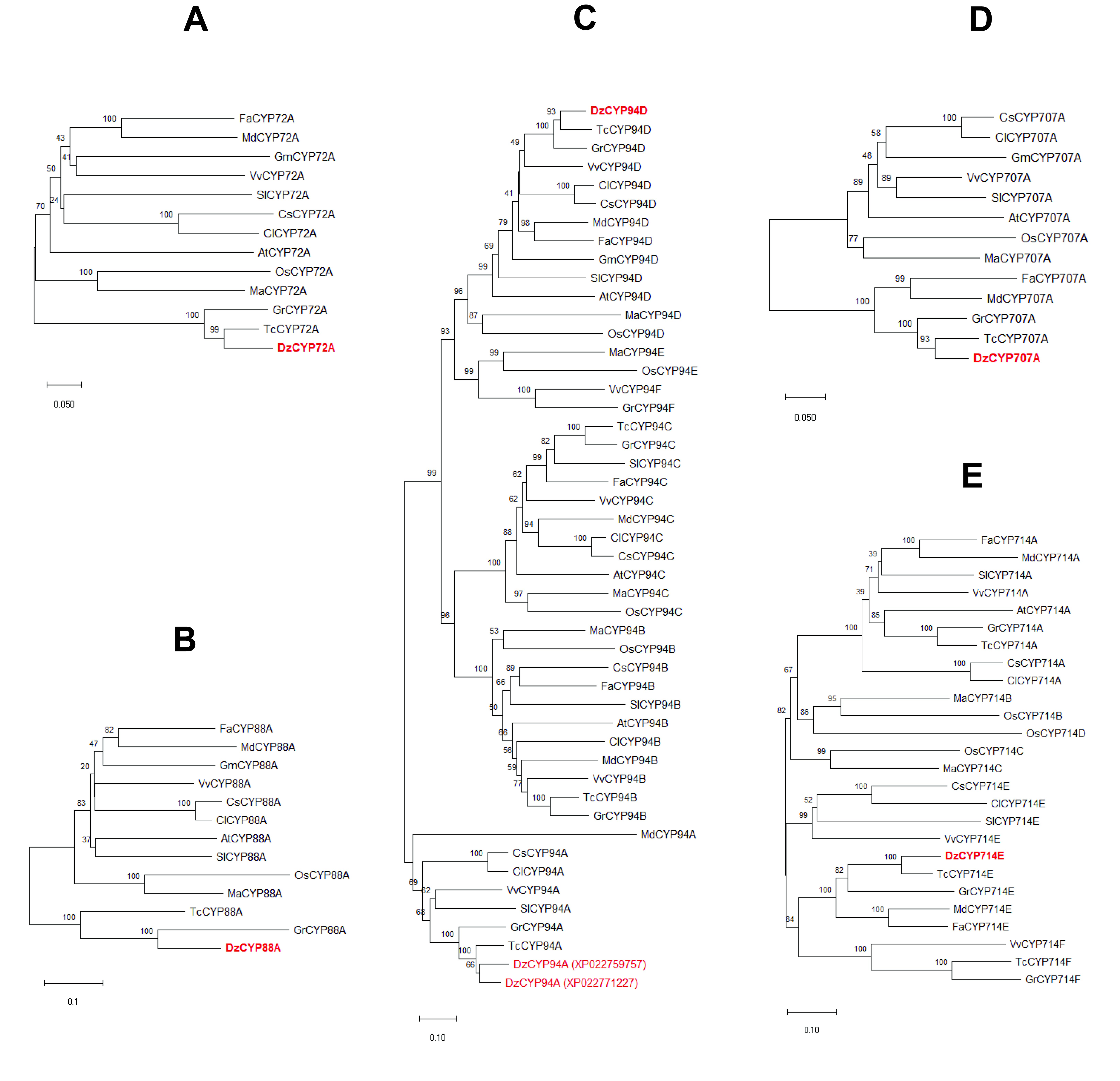

Supplement: S4 Fig — (A) CYP72. (B) CYP88. (C) CYPCYP94. (D) CYP707. (E) CYP714. The candidate durian P450s are highlighted in red letters. The tree was constructed by the maximum likelihood method with 100 replicates. The bars in the trees represent protein relationships of the unrooted tree. The list of protein sequences used to construct the tree is presented in S6 Table. Plant abbreviations: At, Arabidopsis thaliana; Cl, Citrullus lanatus; Cs, Cucumis sativus; Dz, Durio zibethinus; Fa, Fragaria ananassa; Gm, Glycine max; Gr, Gossypium raimondii; Ma, Musa acuminata; Md; Malus domestica; Os, Oryza sativa; Sl, Solanum lycopersicum; Tc, Theobroma cacao; Vv, Vitis vinifera. (TIF) [file pone.0260665.s004.tif]
