# Supplementary material for: Genome-wide identification and expression profiling of durian CYPome related to fruit ripening
Source: PLoS One. 2021 Nov 30;16(11):e0260665. doi: 10.1371/journal.pone.0260665 (PMC8631664; doi:10.1371/journal.pone.0260665)
Supplement: S1 Table — (PDF) [file pone.0260665.s005.pdf]

S2 Table. Primer list for RT-qPCR

Gene name	Accession no.	Primer (5′-3′)
CYP71	XP_022718112	F = GTCAAAGAAACCCTCAGACTAC<br>R = CAGCACCAAACGGGATAAA
CYP71AS	XP_022725705	F = CGATCTCGATCAGCTTCAATAC<br>R = GGGAGAATTCTTCTGGGTTATT
CYP72A	XP_022738158	F = CCAAGCTGGGAAATTTGTGT<br>R = GCCAATTTCGATTCAAGCAT
CYP75B	XP_022736366	F = CTGTGAGATCAATGGCTACC<br>R = AGCTCATACCAGCACATATTC
CYP81B	XP_022716967	F = AACCACACCAGCCTTACCTG<br>R = TCATCAGGTTCAGCATCAGC
CYP81D	XP_022725825	F = TCTTCTGCTTCCACACATGC<br>R = ACCCAAATGGCATTAGCTTG
CYP81Q	XP_022740236	F = AGTTTCAAGCCGGAAAGGTT<br>R = TCGCCTCTAATGGCTCAACT
CYP84A	XP_022763185	F = AACCGAGCAGTACGGTATGG<br>R = CCGTTTTCGACTGAACACCT
CYP88A	XP_022738013	F = CTCCCTGGTTTTACGTTCCA<br>R = GCGAACAAGATCAGCAACAA
CYP94D	XP_022764670	F = CACAAGGTTGATTCCGGTCT<br>R = ACTAAGCGTGGCAGCATCTT
CYP707A	XP_022755802	F = GAGGATTGCTGGGAGTTATG<br>R = GTTGAATACCTTCTTGTTCTCTTG
CYP714E	XP_022743116	F = TTGGCAGGCTATGAGACTA<br>R = TCGGAACTGGAGGGTAAA
**DzEF-1α*	XM_022889169	F = GAAACCTTCTCTGCGTACC<br>R = CTCCACACTCTTGATGACAC
***DzACT*	XM_022897008	F = AATGAGCAAAAAGGGTCAGCAC<br>R = GTCTTCAAAGTCAGCAGCCAG

*Durian elongation factor 1 alpha (first reference gene)

**Durian actin (second reference gene)
