# Supplementary material for: Genome-wide identification and expression profiling of durian CYPome related to fruit ripening
Source: PLoS One. 2021 Nov 30;16(11):e0260665. doi: 10.1371/journal.pone.0260665 (PMC8631664; doi:10.1371/journal.pone.0260665)
Supplement: S2 Table — (PDF) [file pone.0260665.s006.pdf]

S3 Table. Comparison of P450 families in durian and selected flowering plants in the reversed order of angiosperm evolution: Brassicaceae, Malvaceae, Rosaceae, Fabaceae, Cucurbitaceae, Vitaceae, Solanaceae, Poaceae, and Musaceae. The column of durian P450 number is highlight in yellow.

CYP family	*Arabidopsis*	Durian	Cotton	Cocoa	Strawberry	Apple	Soybean	Cucumber	Watermelon	Grape	Tomato	Rice	Banana
clan 71													
71	54	34	55	41	24	50	122	27	27	24	101	105	34
73	1	2	2	3	3	2	5	3	5	3	3	4	6
75	1	6	2	2	2	2	12	1	1	11	2	3	9
76	9	14	16	14	4	16	43	8	8	24	43	38	2
77	7	4	6	2	2	3	4	3	3	2	3	2	4
78	6	12	15	6	6	10	19	5	5	7	6	8	13
79	12	8	14	13	3	3	9	6	10	9	11	4	0
80	0	0	0	0	0	0	0	3	3	6	7	0	0
81	18	26	20	26	9	11	31	19	21	21	14	13	10
82	5	21	41	28	9	27	33	13	21	34	16	0	0
83	2	4	13	17	0	0	23	0	0	0	0	0	2
84	2	5	4	3	3	3	6	3	3	3	1	4	4
89	7	13	9	13	3	12	12	10	6	14	6	20	5
92	0	1	5	2	4	5	2	3	3	6	13	14	4
93	1	7	5	7	6	4	36	2	2	4	1	7	1
98	3	2	2	1	2	6	3	1	1	1	6	3	2
99	0	0	0	0	0	0	0	0	0	0	0	2	0
701	1	1	3	1	2	4	2	1	1	1	1	4	1
703	1	1	1	1	1	1	2	1	1	1	1	1	1
705	26	0	0	0	0	0	0	0	0	0	0	0	0
706	7	8	29	9	7	5	7	5	6	9	17	2	2
712	2	3	4	6	1	0	3	5	5	2	1	0	0
723	0	0	0	0	0	0	0	0	0	0	0	3	0
736	0	14	14	10	4	20	27	14	5	8	25	0	0
clan 51													
51	2	3	2	1	1	2	4	1	1	2	4	12	2
clan 72													
72	10	10	10	8	10	12	30	9	7	22	43	17	17
709	3	2	0	1	0	0	0	0	0	1	0	14	4
714	2	17	7	10	7	11	18	3	3	6	3	6	3
715	1	4	3	1	2	1	7	2	2	1	2	4	2
721	1	2	4	2	1	5	8	3	4	5	2	2	1
734	1	2	3	1	1	1	3	3	2	2	3	5	5
735	2	1	2	1	1	2	5	2	2	1	2	2	2
749	0	21	44	11	16	16	0	3	3	0	3	0	0
clan 74													
74	2	0	8	3	3	3	17	4	4	7	7	6	8
clan 85													
85	2	4	3	2	1	2	12	2	1	2	2	1	4

CYP family	*Arabidopsis*	Durian	Cotton	Cocoa	Strawberry	Apple	Soybean	Cucumber	Watermelon	Grape	Tomato	Rice	Banana
87	2	8	11	9	4	4	4	4	8	7	5	12	2
88	2	5	7	6	4	2	4	12	9	2	5	1	2
90	4	8	6	4	3	6	17	5	7	4	4	5	10
702	6	0	0	0	0	0	0	0	0	0	0	0	0
707	4	15	7	4	7	7	16	6	5	5	4	3	6
708	4	0	0	0	0	0	0	0	0	0	0	0	0
716	2	11	5	8	7	10	11	1	2	15	10	0	0
718	1	1	1	1	1	2	2	1	1	0	1	0	0
720	1	0	0	1	1	1	5	1	1	1	1	0	1
722	1	2	2	3	3	2	4	2	2	1	2	1	1
724	1	4	2	2	2	2	3	1	2	2	2	1	2
728	0	0	3	4	5	5	5	2	2	6	2	14	1
729	0	0	0	1	3	2	0	0	0	0	0	2	0
733	0	1	3	1	1	1	7	1	1	1	1	1	1
clan 86													
86	11	11	11	5	6	11	35	5	5	6	6	9	10
94	7	12	14	8	8	15	21	6	7	9	18	24	22
96	15	10	15	14	0	0	14	8	6	5	12	12	8
704	3	8	6	13	12	8	20	3	3	6	27	6	9
clan 97													
97	3	3	3	3	3	5	16	3	3	3	4	3	2
clan 710													
710	4	2	5	1	0	1	19	1	1	1	1	6	3
clan 711													
711	1	1	1	1	1	4	6	1	1	1	2	5	2
clan 727													
727	0	1	1	1	0	1	1	1	1	1	0	1	3
