# Supplementary material for: Genome-wide identification and expression profiling of durian CYPome related to fruit ripening
Source: PLoS One. 2021 Nov 30;16(11):e0260665. doi: 10.1371/journal.pone.0260665 (PMC8631664; doi:10.1371/journal.pone.0260665)
Supplement: S3 Table — (PDF) [file pone.0260665.s007.pdf]

S7 Table. Normalized Ct values of the genes in durian arils cv. Monthong control (natural ripening), 1-MCP treatment, and ethephon treatment.

**Treatment**	**Normalized Ct value**				
	DzCYP72A	*DzCYP88A*	*DzCYP94D*	*DzCYP707A*	*DzCYP714E*
Control 1	7.35	3.76	10.79	8.48	6.70
Control 2	6.99	4.70	11.08	7.33	6.22
Control 3	7.59	3.56	9.88	6.67	7.29
Control 4	7.61	5.37	10.52	8.21	6.11
Control 5	7.20	3.47	9.78	7.67	7.16
MCP 1	9.00	9.68	9.69	9.25	11.88
MCP 2	7.88	6.34	12.59	9.28	11.18
MCP 3	7.80	7.94	10.33	10.81	10.90
MCP 4	7.15	7.46	10.13	8.91	10.73
MCP 5	7.15	7.82	10.69	9.56	13.03
Ethephon 1	8.11	4.42	9.01	6.92	6.86
Ethephon 2	7.77	3.04	9.56	7.93	7.55
Ethephon 3	6.94	4.07	8.60	7.17	7.12
Ethephon 4	8.57	3.18	9.11	6.93	7.06
Ethephon 5	7.45	3.21	8.77	7.24	6.69
