# Supplementary material for: Genome-wide identification and expression profiling of durian CYPome related to fruit ripening
Source: PLoS One. 2021 Nov 30;16(11):e0260665. doi: 10.1371/journal.pone.0260665 (PMC8631664; doi:10.1371/journal.pone.0260665)
Supplement: S4 Table — The abbreviations N/D indicate no detectable Ct. (PDF) [file pone.0260665.s008.pdf]

S8 Table. Normalized Ct values of the genes in durian arils (two cultivars of each stage). The abbreviations N/D indicate no detectable Ct.

**Cultivar**	**Stage**	**Normalized Ct value**				
		DzCYP72A	*DzCYP88A*	*DzCYP94D*	*DzCYP707A*	*DzCYP714E*
Phuangmanee	Immature1-1	9.80	6.46	8.22	11.89	10.59
	Immature1-2	9.23	6.45	8.15	12.64	12.04
	Immature1-3	9.44	6.52	8.45	12.27	12.55
	Immature2-1	8.27	7.05	8.00	4.88	10.39
	Immature2-2	8.73	6.78	7.99	5.82	11.96
	Immature2-3	8.92	7.11	8.05	6.12	11.17
	Mature 1	7.45	8.54	9.90	4.69	10.32
	Mature 2	7.69	9.18	8.05	4.53	12.43
	Mature 3	7.56	8.50	8.40	4.42	11.38
	Midripe 1	6.59	1.53	5.20	6.19	5.54
	Midripe 2	6.44	1.58	5.11	5.15	4.44
	Midripe 3	6.46	1.55	5.64	5.33	4.58
	Ripe 1	5.19	1.01	3.66	5.62	5.09
	Ripe 2	6.02	1.03	4.38	5.60	6.21
	Ripe 3	5.78	1.64	3.87	5.59	5.72
Monthong	Immature1-1	7.37	7.22	7.83	7.22	N/D
	Immature1-2	9.12	7.11	8.27	7.11	N/D
	Immature1-3	8.05	9.34	8.60	7.88	N/D
	Immature2-1	7.60	8.37	8.93	8.11	N/D
	Immature2-2	9.54	9.50	8.87	8.97	N/D
	Immature2-3	8.57	8.98	8.76	8.24	N/D
	Mature 1	9.14	7.36	8.10	9.48	10.57
	Mature 2	10.53	8.76	7.45	8.42	10.43
	Mature 3	10.63	6.02	9.30	8.86	10.5
	Midripe 1	8.58	3.55	7.82	4.26	6.9
	Midripe 2	8.77	3.66	7.51	4.71	9.3
	Midripe 3	8.33	3.42	7.24	4.44	7.08
	Ripe 1	7.90	2.71	7.66	3.27	6.3
	Ripe 2	6.29	2.75	6.38	3.94	7.06
	Ripe 3	7.64	2.04	7.64	3.88	6.96
