# Supplementary material for: Genome-wide identification and expression profiling of durian CYPome related to fruit ripening
Source: PLoS One. 2021 Nov 30;16(11):e0260665. doi: 10.1371/journal.pone.0260665 (PMC8631664; doi:10.1371/journal.pone.0260665)
Supplement: S5 Table — (PDF) [file pone.0260665.s009.pdf]

S9 Table. Normalized Ct values of the genes in durian arils (four cultivars at ripe stage).

Cultivar	Normalized Ct value				
	DzCYP72A	*DzCYP88A*	*DzCYP94D*	*DzCYP70A*	*DzCYP71E*
Phuangmanee 1	5.20	1.01	3.66	5.62	5.09
Phuangmanee 2	6.02	1.03	4.38	5.60	6.21
Phuangmanee 3	5.78	1.64	3.87	5.59	5.72
Chanee 1	6.49	0.79	4.69	6.54	6.25
Chanee 2	7.68	0.34	4.42	7.15	5.49
Chanee 3	6.86	0.46	5.06	6.37	7.40
Monthong 1	7.90	2.71	7.66	3.27	6.30
Monthong 2	6.29	2.75	6.38	3.94	7.06
Monthong 3	7.64	2.04	7.64	3.88	6.96
Kanyao 1	5.34	2.72	8.60	6.62	8.29
Kanyao 2	5.56	2.39	8.34	6.95	7.58
Kanyao 3	5.26	2.72	9.17	6.66	7.55
