# Supplementary material for: Genome-wide identification and expression profiling of durian CYPome related to fruit ripening
Source: PLoS One. 2021 Nov 30;16(11):e0260665. doi: 10.1371/journal.pone.0260665 (PMC8631664; doi:10.1371/journal.pone.0260665)
Supplement: S6 Table — (PDF) [file pone.0260665.s010.pdf]

S8 Table. List of proteins used for construction of phylogeny of selected plant cytochrome P450s involved in phytohormone biosynthesis.

Plant names	Protein names (Protein ID)
Arabidopsis thaliana	CYP72A (NP188080), CYP88A (NP001320198),<br>CYP94B (NP001331365), CYP94C (NP180337), CYP94D (NP174713),<br>CYP707A (CAA16713), CYP714A (NP568463)
*Citrullus lanatus**	>ClCYP72A<br>MWSVAATISIWIILVVLLLLGCKFVNWIWLKPKKLEKVLREQGFA<br>GNSYRILYGDLKFRAAMREQALSKPMNFSNDIAPRVIPSIHHTIQN<br>YGKNSFMWLGPIPRVHIMDPEQLKIVFSLINDFQKPTINPFARLLA<br>DGILNHEGPKWVKHRKIINPAFHLQKLKDMVPAFYHSCNEMISK<br>WENMVFVEESCELDVMPYLQNMTADVISRTAFGSSYEKGKKIFK<br>LQTELAELVIQSTLGVYIPGWRFLPTKSNNKMKEISNEITTLILGIM<br>NEREKSMKAGEAIQTDLLSILMESNLNEIKQHGNNKDIGMSIEDVI<br>AECKLFYIAGQETTATLLVWTMVLLSSYSEWQEQARAEVFEIFG<br>NKKPDYDGLSQLKVVTMILNEVLRLYPPVSMFGRFVKKETRLGK<br>LTLPAGVMLALPTILIQRDPELWGEDANEFKPERFSKGVSKATKN<br>PSAFVPFGWGPRICIGLNFAMIEAKLALSMILQRFSFELSSSYTHAP<br>TVTITTQPQHGAHIILHKL<br>>ClCYP88A<br>MYTAVLAAVLTVAVWVLKNANHWVYETARLGLKRFALPPGDL<br>GWPFIGNMWSFLRAFKSPYPDSFMDSFFSRYGNTGMYKAFMFGS<br>PSVIVTSAEACKRVLNDDEAFGSGWPLSTMKLIGEKSFIGISYQEH<br>KRLRRITAAPVNGYDALATYLTYIEKIVVSSLEKWASMGQIEFLT<br>HLRKLTFKIIMYIFLSSETEAIMEALEREYTELNHGVRAMAINIPGF<br>AYYKALKARKNLVATFGSIVRGRRKERERKNNLRTKRRDMMDA<br>LLDAEDEDGRRLSDEEIIDILIMYLNAGHESSGHTMMWATIFLQQ<br>HPQFLQKAKAEQEEIVKKRPPGQKGLTLKEVREMTYLSKVIDETL<br>RVVTFSLTVFREAKHDVKISGYTIPKGWKVLVWFRSVHHDPETY<br>PNPKEFNPSRWDDFTPKAGSFLPFGAGSRLCPGNDLAKLEISIFLH<br>YFLLSYKMERVNPKSPVRYLPHSRPKDNCLARIKRLD<br>>ClCYP94A<br>MLSPLLLFFILFLLPLLFFIFIFTKTPNSPFTPPIKLPKSYPLVGSFFAV<br>FANRRRRLQWLSDVLQVSPAATFTLHRPFGQRQIFTANPAVVQHI<br>LKTHFHIYQKGDSFRSVFSDFLGDGIFNADGESWKFQRQVSSHEF<br>TTKSLRKFVETVVDAELSDRLVPVLYTAAADCSVLDFQDVLQRF<br>AFDNVCKIAFGYDPAYLSPSFGQSKFAKAFEDAVRISSLRFQSLIPF<br>VWKLKKFLDIGSEKRLRIAIAEVRGYANNIINDKKTELKTNSSIPS<br>VDLLSRFLTSGHSDHNFITDIIISFILAGQDTTSAALTWFFWLLAKY<br>PQVEIRILEEISQKTEDLFGYDEVKDLTYTHAALCESMRLYPPVPV<br>DGKQAAADDVLPDGTAVRKGERVAYHPYAMGRMEAIWGKDW<br>AKFRPERWLESDSGDEATEVKWRFVGRDNYTYPVFQAGPRICLG<br>KEMAFLQMKRMVAGILKRFRVVPAAAEEGVEPRFVQYMTAKM<br>EGGFPVRIEVREGSE

Plant names	Protein names (Protein ID)
	>ClCYP94B<br>MIYFSVSFLLLSSSLWAFFLLYLCISKFYCFSRKRFYYSPPSYPFFG<br>CLISFYKNRRRLLAWYTDLLSDSPTQTIVVHRLGSRRTVVTANPA<br>NVEHMLKTNFINYPKGKPFTDILGDLLGCGIFNVDGDLWSSQRKL<br>ASHAFSAKSLREFVVKTLEDEVHLRLIPLLSNAARTNAVIDLQDV<br>LGRLAFDTVCKVTLGTDEQCLDMSRPIPEIVNAFDTATGISAKRAI<br>APLFLTWKAKRMLNVGSEKKLKEAVQMVQKWVSNIIRNKNNNT<br>LHNHNQTHNNDLLSRLLSTNHDEEVVRDMIISFIMAGRDTTSAA<br>MTWLFWLLTKHPKAEQAIINEATSLSDDSKTMSLGYGYEELKDM<br>KYLKACLCESMRLYPPVAWDSKHAAAADILPDGTPVRKGDRVT<br>YFPYGMGRMEEVWGKDRLEFKPDRWFQNGELKTVSPFKFPVFQ<br>AGPRMCLGMEMAFIQMKYVVATVLNRFEFRPASENSPVFVPLLT<br>AHMAGGLRVYVRERIEQKSTMW<br>>ClCYP94C<br>MSGSELLFSLPTAISLLFFSFTAAFTIFSFSLYILRLNPCCNCCVCRS<br>YLSSSWSFSFPNLSDWYTHLLSLSPTATIHLHVLSNIVTANPDNVQ<br>HILKSNFHNYPKGKPFSSILGDLLGHGIFNVDGHSWRFQRKMASL<br>ELGSLSLRSHAFEILTTQIQTRLVPVMKDVGEVDLQDVFRRFSFD<br>NICRFSFGLDPGCLRLCLPISEFAVAFDLASRLSAERAMAASPIMW<br>KIKRVLRVGSERKLREAIKMVDGLAMEVIRQRREMGFSHRNDLL<br>SRFMASTNDDRYLRDIVVSFLLAGRDTVASALTSLFWLLSQNPEV<br>ETKIISESDRIMGPDRDTVPNFDNLKDMHYLQAAVYENMRLFPPV<br>QFDSKFAEEDDILPDGTFVQKGTRVTYHPYAMGRMDRIWGPDCL<br>EFKPERWLKNGYFAPENPFKFPVFQAGLRVCLGKELAVMDVKC<br>VAVVLIRKFKIRLAEADRVARFAPGLTASWRGGLPVRIEERTTC<br>>ClCYP94D<br>MDISVLSVLTLTPFIFLTFLSLSLYLLFFTSPNTKSKPHQGFKHFPL<br>VGTLPLFLLNRHRFLDWSTEVLKSCRTNTAVFKRPGKVHGVITA<br>NPLVVEHILKTQFENYPKGERFISLLEDFLGRGIFNSDGEIWKVQR<br>KTASYEFNTKSLRNFVMENVRVEVQSRLLPIFGKACETERILDLQ<br>DVLERFAFDNVCKLAFNYDPACLGGDGTAAAEFMRAFEDAATL<br>SSGRFMYAFPGLYKVKKFLNIGSERSLKEAIAIVHKFADDIISSRM<br>EEKKRTQIEKDQDLLSRFMGDENNSPEFLRDIIISFILAGRDTTSSA<br>LTWFFWILSSRPDVEQKILTELQTIRSRTHKEIGEMYGFDQLRDM<br>QYLQAALSETLRLYPPVPVDTKACRNDDVLPDGTFIGKSWFVTY<br>HTYAMGRMESIWGKNYGEFSPERWLQNGVCKTESPFRFPIFHAG<br>PRMCLGKDMAYIQMKSIAAAVIEKFEVEMVEKKKTPKHLLSLTL<br>RMENGLQVMVKKRDRSMPI<br>>ClCYP707A<br>MFAFSVLLLLLVFLLFFHFLFKFSAPALRKLPLPPGSMGWPYLGET<br>LQLYSQDPNVFFASKKKRYGPIFKSHILGYPCVMVSSPEAAKFVL<br>VTKAHLFKPTFPASKERMLGKNAIFFHQGDYHAKLRRLVLRTFM<br>PEAIRNIVPSIESIAKNTVQSWDGQLINTFQEMKMFAFEVSLLSIFG<br>KDEALYFEDLKRCYYILEKGYNSMPINLPGTLFHKAMKARKELA

Plant names	Protein names (Protein ID)
	HILNKILSTRREMKRDHNDLLGSFMGEKEGLTDEQIADNVIGLIFA<br>ARDTTASVLTWILKYLGENPSVLQAVTAEQEAIMKQKQSGDDNL<br>TWADTKNMPITSRVIQETLRMASVLSFTFREAVEDVEFDGYLIPK<br>GWKVLPLFRNIHHSPENFPQPDKFDPTRFEVAQKPNTYMPFGNGT<br>HSCPGNELAKLEMLVLLHHLTTKYRWNVVGAQEGIQYGPFALPL<br>NGLPIKIFLKK<br>>ClCYP714<br>MEGSIIGGFVSVFPVAVAVTVVAILVHLYFYGAWRRTAEVRRKL<br>RAQGVIGPPPSVLYGNLPEMQKIQLQTAAMASPPHGASAIIAHDY<br>TSTLFPYFVEWRKQYGPLYTYSTGMRQHLYANKVELVKDLNFSN<br>NINLGKPFYVTKKLAPILGHSVVRSNGAVWAVQRKIIAPEFFMDR<br>VRAMVGLMADAALCLIDKWESRIGDGATAEIEVDEDLRGFSADV<br>ISRACFGSWYEKGKEIFSKLRDLQRLVSEGSFLFGYISLSDRFFQRS<br>KHRRIKKLEKEIESLIWETVQQRQKECSKTSSSEKDLLQLIMEATT<br>NDPNIGANDSSKNFIVDNCKSIYFAGHESTAVAATWSLMLLALHP<br>EWQDCIRSEFAHACPDGHPDMAVISQLKSVSMVVHETLRLYPPA<br>AFVARETFAEMQLGNVVVPKGVCLWTLIPSLHREVEIWGEDANK<br>FKPERFENGIAKACKFPQAYVPFGAGPRLCLGKNFALVELKIIISL<br>VVSKFRFSLSPEYRHCPSYRMIVEPANGVKIVFQRL<br>>ClCYP714E<br>MAVMVLSVLAAVSFFIFLHLFESLFLKPKRLRSKLRNQGIDGPSPS<br>FLLGNLPEIKNIRAIKSHALTTTEGDDSIAHGWPSNLLPHLEHWRN<br>RYGPKFVYSSGTVQILCITDVEMVKEIGLSTTLNLGKPAHLSKDR<br>GPLLGLGILASSGPIWVHQRKTIAPELYLDRVKDMTSLMVESVKS<br>MIKSWETIVENDGGQSELNVDSYFRTLSADIISKAWVFKLSCPKK<br>TLVFLGSGIYISIYMGDVILFDLEKKSYAMYLPTKDNREIWKLEKE<br>IESMVLEVVKKRIKQCSKEKDLLQIIIEDAKCLDVEGKSLKISGDK<br>FIVDNCKNIYFAGHETTAITASWCLMLLAKHPDWQARVRSEVLE<br>CCQDGTLDAETIKKMKTLTMVIQETLRLYPPGVFVTREALEDLRF<br>KNLRIPKGMHHNVQLWGPDVLSFNPQRFSNGILKACKNPQAYIPF<br>GVGPHICAGQHFAMVELKVIVSLIVSKFEFSLSPSYNHSPAFSLVV<br>EPKNGVLLHLRNLSSFSL
Cucumis sativus	CYP72A (XP011659939), CYP88A (XP011656712), CYP94A (XP011658957), CYP94B (XP011651558), CYP94C (KGN64915), CYP94D (XP004136768), CYP707A (XP004150544), CYP714A (XP004146466), CYP714E (XP004138935)
Durio zibethinus	CYP72A (XP022738158), CYP88A (XP022738013), CYP94A (XP022759757), CYP94A (XP022771227), CYP94D (XP022764670), CYP707A (XP022755802), CYP714E (XP022743116)
Fragaria ananassa	CYP72A (XP004294188), CYP88A (XP011469589), CYP94D (XP004299232), FaCYP94C (XP004305353), CYP94B (XP004303976), CYP707A (XP004295971), CYP714A (XP004307754), CYP714E (XP004294859)

Plant names	Protein names (Protein ID)
Glycine max	CYP72A (XP003531823), CYP88A (XP003546291), CYP94D (ABD97099), CYP707A (XP003534341)
Gossypium raimondii	CYP72A (XP012438179), CYP88A (XP012475722), CYP94A (XP012476259), CYP94B (XP012489010), CYP94C (XP012485820), CYP94D (XP012456972), CYP94F (XP012472918), CYP707A (XP012471298), CYP714A (XP012443451), CYP714E (XP012458489), CYP714F (XP012448130)
Musa acuminata	CYP72A (XP009394609), CYP88A (XP009392783), CYP94B (XP009407042), CYP94C (XP009411963), CYP94D (XP009383332), CYP94E (XP009414953), CYP707A (XP009396959), CYP714B (XP009406861), CYP714C (XP009381346)
Malus domestica	CYP72A (XP008366858), CYP88A (XP028961309), CYP94A (RXH86486), CYP94B (RXH77817), CYP94C (XP008371286), CYP94D (XP008393292), CYP707A (XP008356917), CYP714A (XP008343675), CYP714E (XP008354178)
Oryza sativa	CYP72A (XP015615867), CYP88A (XP015643774), CYP94C (XP015615638), CYP94D4 (XP015616099), CYP94E (XP015621268), CYP707A (XP015627713), CYP714B (XP015645488), CYP714C (XP015619748), CYP714D (XP015637923)
Solanum lycopersicum	CYP72A (XP004242635), CYP88A (XP004229509), CYP94A (XP004242690), CYP94B (XP004233587), CYP94C (XP015086840), CYP94D (XP004249121), CYP707A (NP001362844), CYP714A (XP004235212), CYP714E (XP004229723)
Theobroma cacao	CYP72A (EOX99507), CYP88A (XP017972617), CYP94A (XP007050381), CYP94B (EOY22465), CYP94C (EOY08182), CYP94D (EOY19355), CYP707A (EOY33987), CYP714A (EOY29240), CYP714E (EOY19021), CYP714F (EOY34111)
Vitis vinifera.	CYP72A (XP002263652), CYP88A (XP002264215), CYP94A (CAN75729), CYP94B (XP002279981), CYP94C (XP002264897), CYP94D (XP002278009), CYP94F (RVX19985), CYP707A (NP001267981), CYP714A (XP002266768), CYP714E (XP002269307), CYP714F (XP002273288)

* *Citrullus lanatus* cytochrome P450s are exclusively available on P450 homepage (https://drnelson.uthsc.edu/), without NCBI accession numbers.
