# Supplementary material for: Genome-wide identification and expression profiling of durian CYPome related to fruit ripening
Source: PLoS One. 2021 Nov 30;16(11):e0260665. doi: 10.1371/journal.pone.0260665 (PMC8631664; doi:10.1371/journal.pone.0260665)
Supplement: Attachment — Submitted filename: Response to Reviewers.docx [file pone.0260665.s012.docx]

**Table of response to the editor’s comments**

| **Editor’s comments** | **Authors’ responses** |
| --- | --- |
| 1. Table S7a, S7b, S7c should be separate supp tables (S7, S8, S9). | Following the Editor’s comment, the tables S7a-S7c have been changed to S7 Table, S8 Table, and S9 Table, respectively. |
| 2. Fig 1 legend (L223 track changes version) – please reword to clarify what you mean by “bars in the trees” | Following the Editor’s comment, we have adjusted the sentence to " The scale bars in the circular trees represent the number of amino acid substitutions per site" instead (line 223). |
| 3. Fig 4  Bar charts are not logical with these x axes (esp. as y intercept not at 0) – please change to an appropriate alternative (e.g. scatter charts, box plots, violin plots)  Please make the x axis labels consistent (can also remove unnecessary repetition) – e.g. “Relative Expression (log FC)” together with an explanation of calculation in the methods would be sufficient. | Following the Editor’s comment, figure 4 has been changed. The labels of the y-axis and the legend of Fig 4 have been rewritten to be more concise. |
